# A large intrathoracic goiter with tracheal stenosis: Complete resection using a robot‐assisted thoracoscopic approach

**DOI:** 10.1111/1759-7714.14470

**Published:** 2022-05-13

**Authors:** Ryuji Nakamura, Katsuhiro Okuda, Kensuke Chiba, Takuya Matsui, Risa Oda, Tsutomu Tatematsu, Keisuke Yokota, Ryoichi Nakanishi

**Affiliations:** ^1^ Department of Oncology, Immunology and Surgery Nagoya City University Graduate School of Medical Sciences Nagoya Japan

**Keywords:** complication, large intrathoracic goiter, minimally invasive surgery, robot‐assisted thoracoscopic surgery, tracheal stenosis

## Abstract

Growing intrathoracic goiters may compress surrounding organs and deteriorate the cardiopulmonary function. Treating such cases requires carefully considering how to maintain oxygenation and resect the tumor with minimal invasiveness without complications. We herein report a surgically resected case of a large intrathoracic goiter‐compressed trachea extending from the right lower pole of the thyroid gland to the carina. We secured the airway by intubation preparing for extracorporeal membrane oxygenation and successfully performed surgical complete resection using a robot‐assisted thoracoscopic and cervical approach. Intrathoracic goiter is a tumor with abundant neovascularity, and the right vagus nerve is displaced in the thoracic cavity, but a robot‐assisted thoracoscopic approach using CO_2_ insufflation improved visualization at the narrow apex area of the thoracic cavity. Robot‐assisted thoracoscopic surgery is a useful surgical procedure enabling safe and minimally invasive surgery without recurrent laryngeal nerve palsy or tracheal injury for intrathoracic giant goiters extending into the thoracic cavity.

## Graphical abstract

Chest computed tomography shows the trachea stenosed by the goiter (a). Coronal (b) and axial (c) sections show the tumor extending beyond the level of the carina.

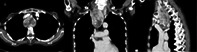

## INTRODUCTION

Intrathoracic goiters requiring a thoracic approach for resection are relatively rare but can compress the surrounding organs and obstruct the cardiopulmonary function.^1^, ^2^, ^3^ Recently, a case of a mediastinal mass inducing life‐threatening airway obstruction and requiring extracorporeal membrane oxygenation (ECMO) during general anesthesia to maintain oxygenation was reported.^4^ Intrathoracic goiters are usually resected through a cervical approach, but the approach should be determined carefully based on the location and size. An additional thoracic approach may be required to prevent injury of large vessels and nerves, so an adequate, low‐invasiveness approach must be considered for each case.

## CASE PRESENTATION

**FIGURE 1:**
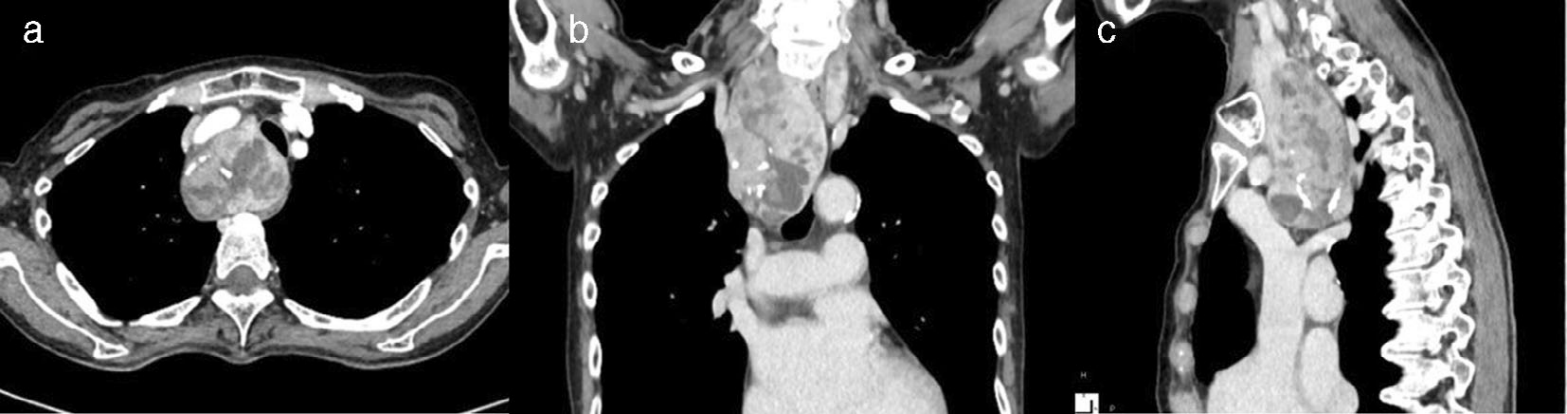
Chest computed tomography showed the trachea stenosed by the goiter (a). Coronal (b) and axial (c) sections showed the tumor extending beyond the level of the carina

**FIGURE 2:**
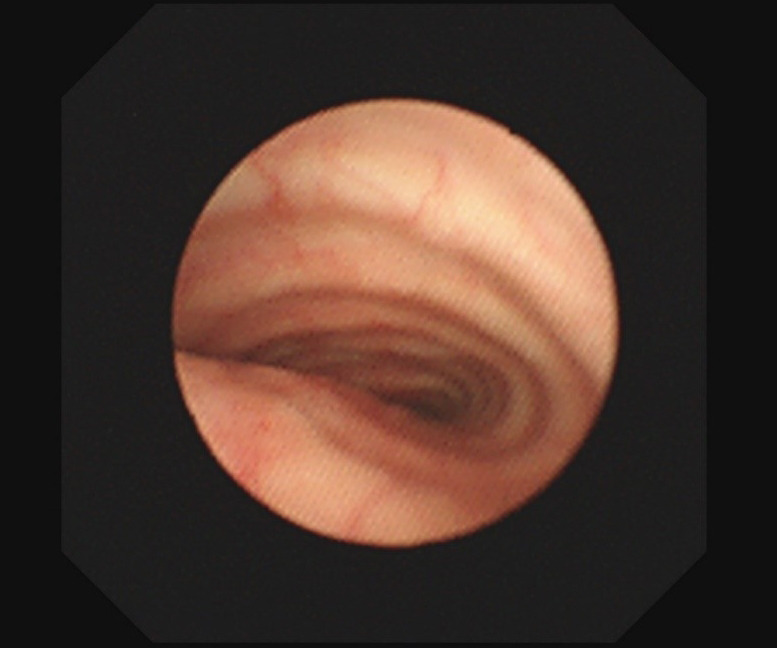
Bronchoscopy revealing that tracheal stenosis was caused by the goiter and the tracheal mucosa was intact

A 73‐year‐old woman was admitted to our hospital for ulcerative colitis. Chest X‐ray on admission showed a widened mediastinum and shifted trachea, so she was referred to general thoracic surgery. Pulmonary function tests showed a forced expiratory volume in 1 s (FEV1) of 1.19 L, forced vital capacity (FVC) of 2.09 L, peak expiratory flow (PEF) of 1.83 L, and %PEF of 27.2%. Chest computed tomography (CT) revealed a mediastinal mass (54 × 41 × 96 mm) extending from the right lower pole of the thyroid gland to the carina, compressing the trachea (Figure 1a–c). She had no complaints of dyspnea, but the tracheal lumen was just 3.5 mm. Preoperative bronchoscopy revealed intact tracheal mucosa, and a 4‐mm bronchoscope could pass through the stenosed lesion (Figure 2). Thyroid tumor resection was planned to avoid complete airway obstruction. We prepared to use ECMO before starting general anesthesia, predicting difficulty maintaining oxygenation during intubation.

First, each femoral vein was cannulated using a 4‐Fr sheath to prepare for veno‐venous ECMO in a room with a fluoroscope for angiography. We then administered general anesthesia and intubated her with a 5.5‐Fr tracheal tube via bronchoscopy. The blocker catheter was palced at the right main brounchus using a 2 mm bronchoscopy. The respiratory condition was stable on one‐lung ventilation in the left decubitus position, so she was transferred to the operating room with a da Vinci robot system.

**FIGURE 3:**
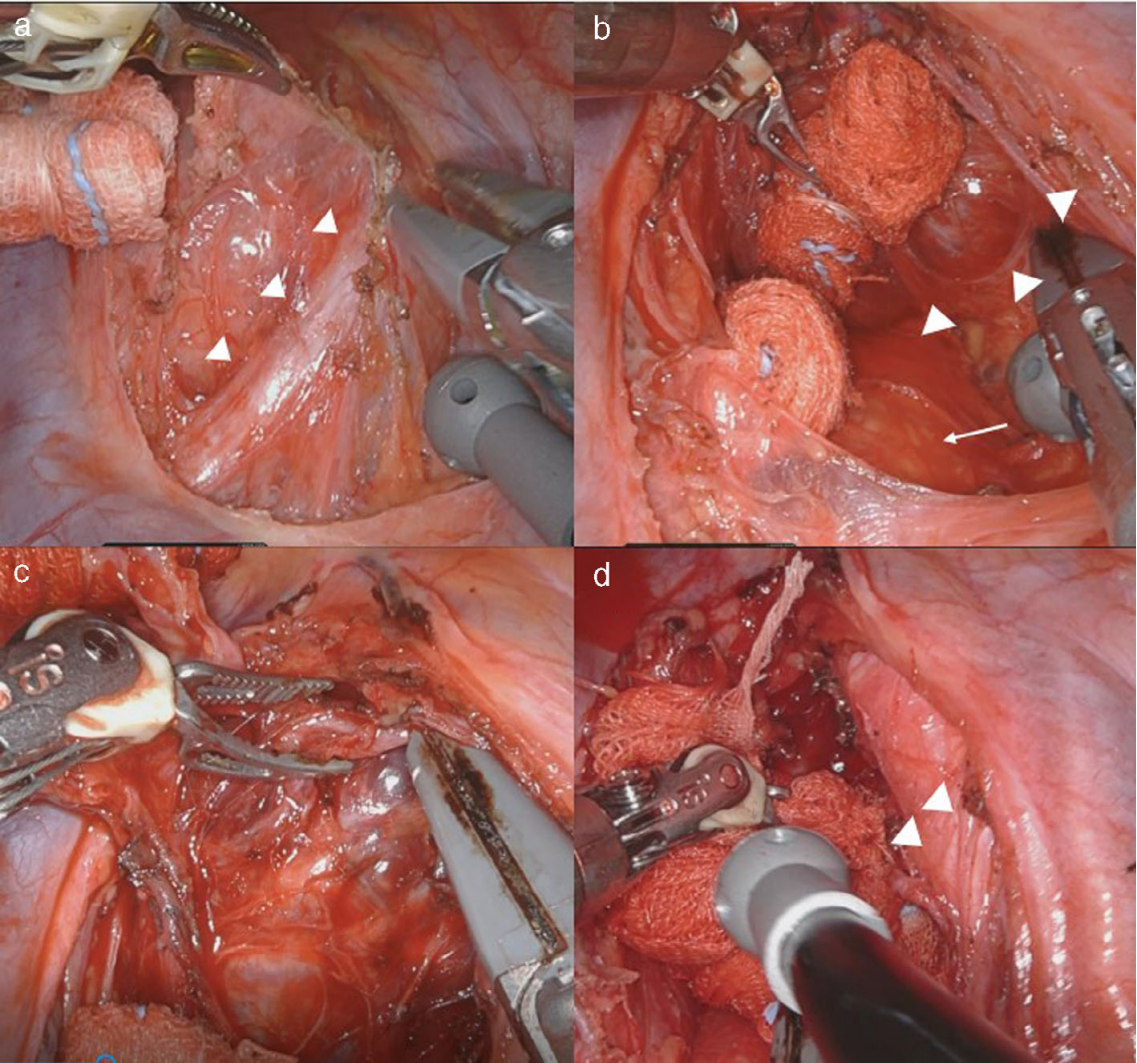
The tumor occupied the superior mediastinum, with the vagus nerve lying over it. The arrowhead indicates the vagus nerve (a). The tumor lacked direct infiltration of the surrounding organs. The arrowhead and arrow indicate the tumor and trachea (b). The tumor was hypervascular (c). The arrowhead indicates the right recurrent nerve (d)

**FIGURE 4:**
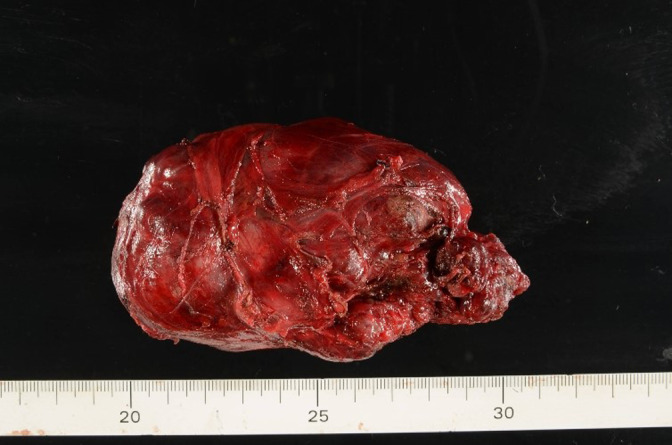
Surgical specimen of the tumor showing that it was completely encapsulated

We started the operation via the thoracoscopic approach. We made three ports at the right 8th intercostal space (the first port on the middle axillary line, the other two ports each 6 cm apart on the dorsal site from first port) and one port on the anterior axillary line at the 6th intercostal space. An assist port was made on the anterior axillary line at the 3rd intercostal space, with CO2 insufflation (8 mmHg) into the pleural cavity. The tumor occupied the superior mediastinum, with the vagus nerve lying over it (Figure 3a). The tumor lacked direct infiltration of the surrounding organs but was hypervascular (Figure 3b,c). A careful surgical approach with preservation of the right vagus nerve, recurrent laryngeal nerve, and trachea was needed (Figure 3d). The right lower pole of the thyroid was confirmed, and surgery via the thoracoscopic approach was completed. Finally, the right thyroid gland and tumor were removed via the cervical incision (Figure 4). We confirmed the improved trachea stenosis and no vocal cord paralysis by bronchoscopy.

The operation time was 357 min, and blood loss was 151 ml. A microscopic examination showed a benign adenomatous goiter. She was treated for ulcerative colitis and discharged without complications on postoperative day 19.

## DISCUSSION

Recently, several researchers have described cases of ECMO for mediastinal masses resulting in life‐threatening airway obstruction during general anesthesia.

Blank et al. recommended using ECMO when patients had dyspnea before surgery or the tumor obstructed over half of the trachea on computed tomography.^5^ The present patient had no symptoms, but the trachea cross‐sectional area at the narrowest lesion had decreased from 251 to 61 mm^2^ (stenosis down to 24%), so we prepared ECMO. ECMO is effective for providing respiratory support to patients with potential airway collapse, but its issues include friability and high invasiveness. We thus need to consider the indication for each case.

Regarding the surgical approach for intrathoracic goiter, some researchers claim that excision is possible only via the cervical approach,^6^, ^7^, ^8^ while the others have reported that a thoracic approach was needed in certain cases, such as goiters located in the posterior mediastinum, extending more caudally than the aortic arch or carina, and located over 5 cm from the thoracic inlet.^8^, ^9^, ^10^, ^11^, ^12^, ^13^, ^14^ There is thus no obvious consensus on the need for an additional thoracic approach. Furthermore, the capsule of the tumor was friable, making it unsafe to perform a blind procedure at the caudal side from the cervical incision. The retro‐tracheal tumor extended to the carina (7.5 cm from the thoracic inlet), so we considered the thoracic approach to be suitable. Because the patient had hyperthyroidism and uncontrolled ulcerative colitis, we performed robot‐assisted thoracoscopic surgery to avoid excessive invasion.

The robotic approach facilitates precise dissection around the vascular and nervous structures because of its three‐dimensional high‐vision and multidirection of the articulated arms.^15^, ^16^ These characteristics allow for accurate surgical resection to be performed in narrow anatomical spaces, such as at the apex of the thoracic cavity. In the present case, a huge intrathoracic goiter extending to the carina was resected without difficulty by a combined robot‐assisted thoracoscopic and cervical approach.

## CONFLICTS OF INTEREST

The authors have no conflicts of interest to declare.
